# A Sting in the Tale: Spurious Erythrocytosis Following Bee Stings Mimicking Gaisböck’s Syndrome

**DOI:** 10.7759/cureus.68255

**Published:** 2024-08-30

**Authors:** Manjeet Kothari, Harshitha Reddy, Sunil Kumar, Harsh Babariya, Tejas Nehete

**Affiliations:** 1 Medicine, Jawaharlal Nehru Medical College, Datta Meghe Institute of Higher Education and Research, Wardha, IND

**Keywords:** angioedema, hematocrit, erythrocytosis, bee sting, gaisböck's syndrome

## Abstract

All around the world, bee stings are rather common and can be deadly, leading to the development of angioedema. Bee venom comprises a variety of physiologically active compounds and enzymes that can occasionally be lethal, producing both local and systemic reactions. Multiple stings can result in toxic shock syndrome with systemic symptoms, while simple stings might induce moderate reactions such as skin rash and urticaria, and sometimes even anaphylaxis. Angioedema and local plasma extravasation are caused by localized changes in the permeability of postcapillary venules and subcutaneous or submucosal capillaries in response to mediators such as histamine and bradykinin.

Gaisböck's syndrome is a disorder marked by an apparent increase in red blood cell mass without an actual increase in red cell volume. It is sometimes referred to as stress polycythemia or pseudopolycythemia. This disorder frequently results from a decrease in plasma volume, which causes the concentration of red blood cells to seem higher than usual. The increase in diastolic blood pressure appears to be connected to the decrease in plasma volume. There is still uncertainty about the nature and classification of the syndrome, and the abundance of synonyms exacerbates the confusion. Gaisböck's syndrome has a complex pathophysiology and multiple clinical problems, which improve our understanding of the causes of erythrocytosis during patient examination.

This report features a 47-year-old man who had multiple bee stings and developed relative erythrocytosis. After receiving treatment, the patient was reportedly doing well.

## Introduction

Hymenopteran insects, including hornets, wasps, ants, and honey bees, are frequently responsible for unintentional human stings worldwide. Prior allergic reactions may raise the possibility of severe, potentially fatal consequences. Each person will react to bee venom differently, in terms of intensity, length, and frequency of symptoms. The venom of Hymenoptera is made up of a mix of enzymes and physiologically active compounds that can occasionally be lethal and produce both localized and systemic reactions [1].

The term "Gaisböck's syndrome" describes a group of illnesses that Felix Gaisböck, MD, of Innsbruck, Tyrol, Austria, initially identified in 1905 [2]. The stocky, overweight build, suffusion of the eyes, plethoric appearance, tense and anxious personalities, cigarette-smoking habit, vascular sickness, migraines, and facial rubor were the characteristics of these individuals. Further investigation revealed that alcohol, diuretic medication, and physical or psychological stress were additional risk factors that might influence the onset of this illness [2].

Relative polycythemia, in which the red cell mass is within normal limits but the hematocrit is elevated, differs from absolute or real erythrocytosis. The reduced plasma volume may be the cause of the higher hematocrit [3]. A sudden enlargement of the skin, mucous membranes, or both, encompassing the digestive and respiratory systems, is known as angioedema. The swelling is nonpitting, skin-colored, or occasionally erythematous. Usually, swelling recovers in a day or more and disappears without leaving any skin discoloration behind. Angioedema and local plasma extravasation are caused by localized changes in the permeability of postcapillary venules and subcutaneous or submucosal capillaries in response to mediators such as histamine and bradykinin. Urticaria and edema may coexist. Histamine-induced angioedema, also known as allergic angioedema, is a hypersensitive reaction to a variety of substances, including meals, medications, and insect venoms. Bradykinin-induced activation of endothelial cells, which causes vasodilatation and capillary leakage, is thought to be the source of kinin-induced angioedema [4].

Two types of angioedema induced by kinins are recognized: drug-induced and inherited. On the other hand, idiopathic and mixed types exist that are unknown in origin.

## Case presentation

**Figure 1:**
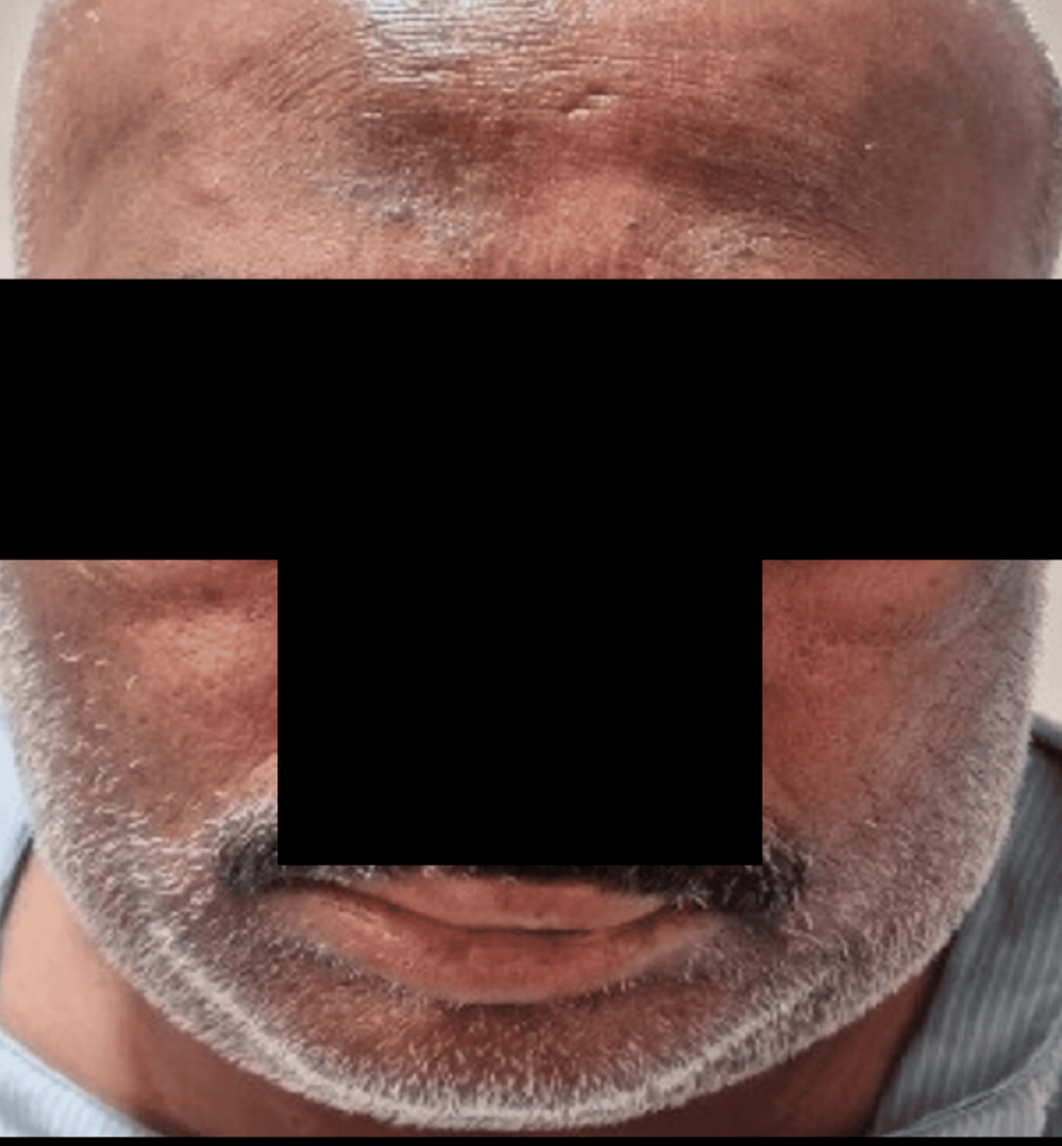
Facial puffiness after multiple bee stings in the patient

A 47-year-old male was brought to the hospital with an alleged history of multiple bee sting bites on the body, and more on the face, while working on the farm one hour ago, which is at a distance of half a kilometer from the hospital. The patient has no history of any comorbidities or previous medical conditions. The patient had complaints of facial puffiness (Figure 1).

**Figure 2:**
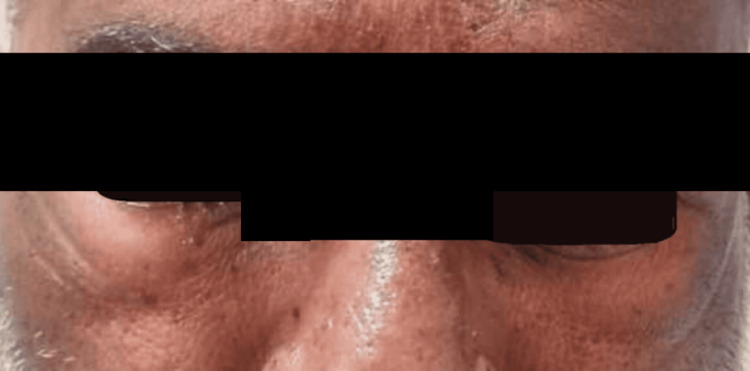
Periorbital edema with redness of eye post bee stings

**Figure 3:**
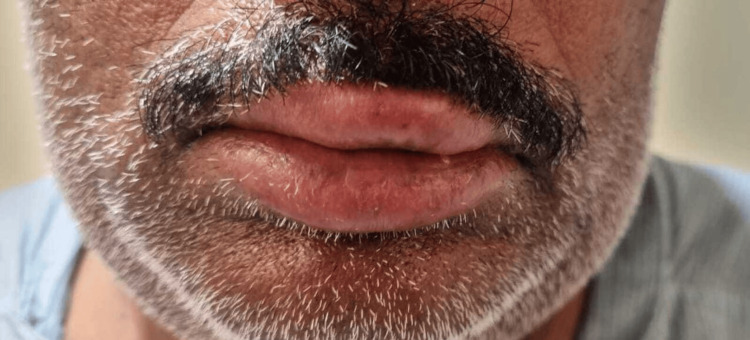
Angioedema post bee stings

Periorbital swelling with redness of the eye (Figure 2), angioedema (Figure 3), difficulty in swallowing, and difficulty in breathing. There are no other complaints of chest pain, palpitations, sweating, cough, or blurring of vision. The patient has no comorbidities.

**Figure 4:**
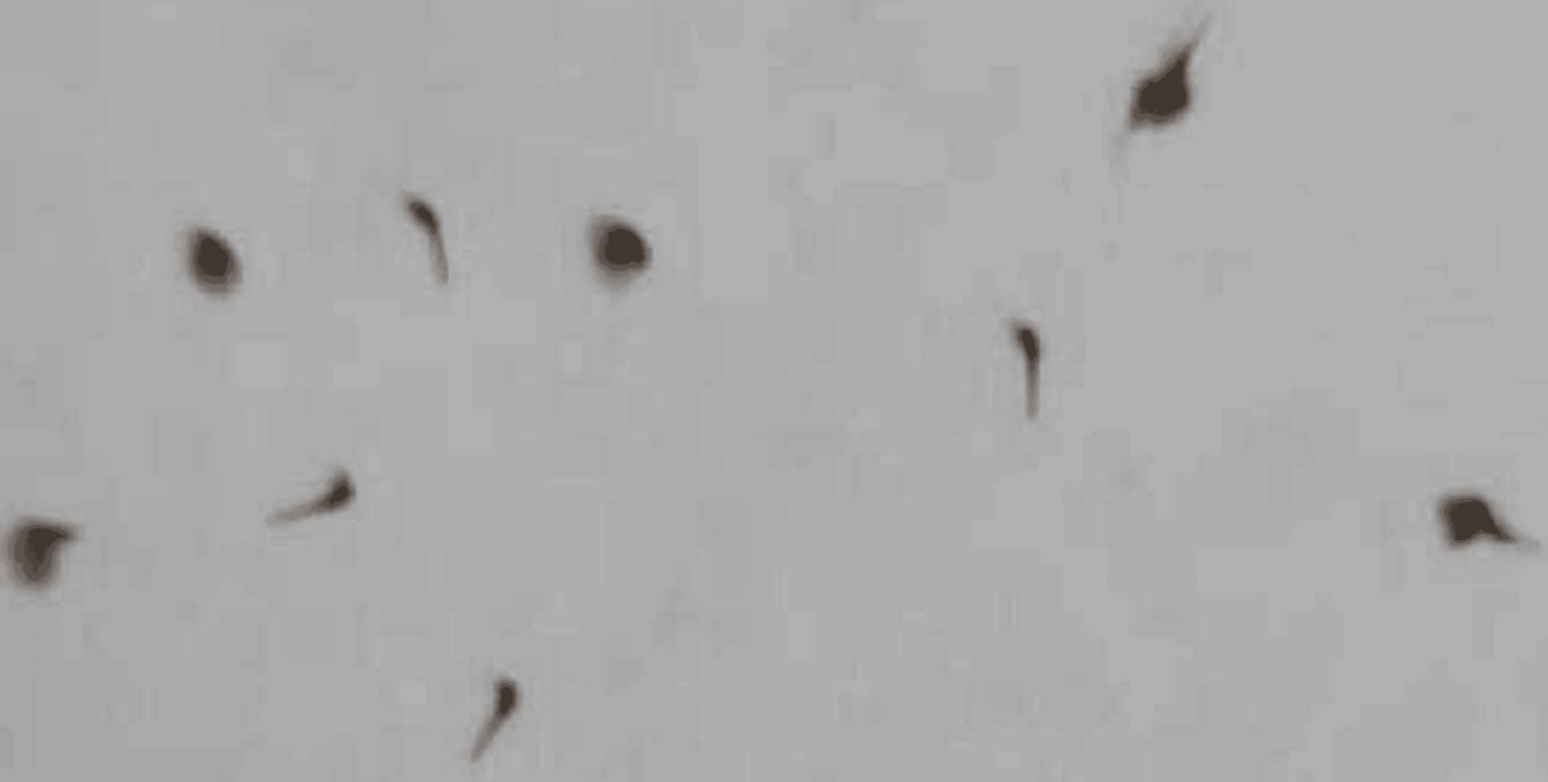
Bee stings removed from the patient's body

Physical examination revealed no pallor, icterus, cyanosis, clubbing, or lymphadenopathy. Blood pressure was 90/60 mmHg, heart rate was 116 beats per minute, and respiratory rate was 24 cycles per minute. All systemic examinations were normal. Carefully, all the bee stingers imbedded in the patient's body were removed (Figure 4).

All laboratory investigations are highlighted in Table 1.

**Table 1:** Laboratory investigations of the patient

Lab investigation	Observed value	Reference of lab
Hemoglobin	19.2	13-15 g/dL
Total leucocyte count	4,600	4,000-11,000/cumm
Platelet	2,10,000	1,50,000-4,50,000/cumm
Mean corpuscular volume (MCV)	87.8	79-100 fL
Hematocrit	60	>45%
Urea	16	9-20 mg/dL
Creatinine	1	0.6-1.2 mg/dL
Sodium	139	137-145 mmol/L
Potassium	3.8	3.5-5.1 mmol/L
Alkaline phosphatase	80	38-126 U/L
Alanine transaminase (ALT)	52	<50 U/L
Aspartate transaminase (AST)	45	17-59 U/L
Total protein	6.2	6.3-8.2 gm/dL
Serum erythropoietin	7.6	2.3-18 mU/mL

**Figure 5:**
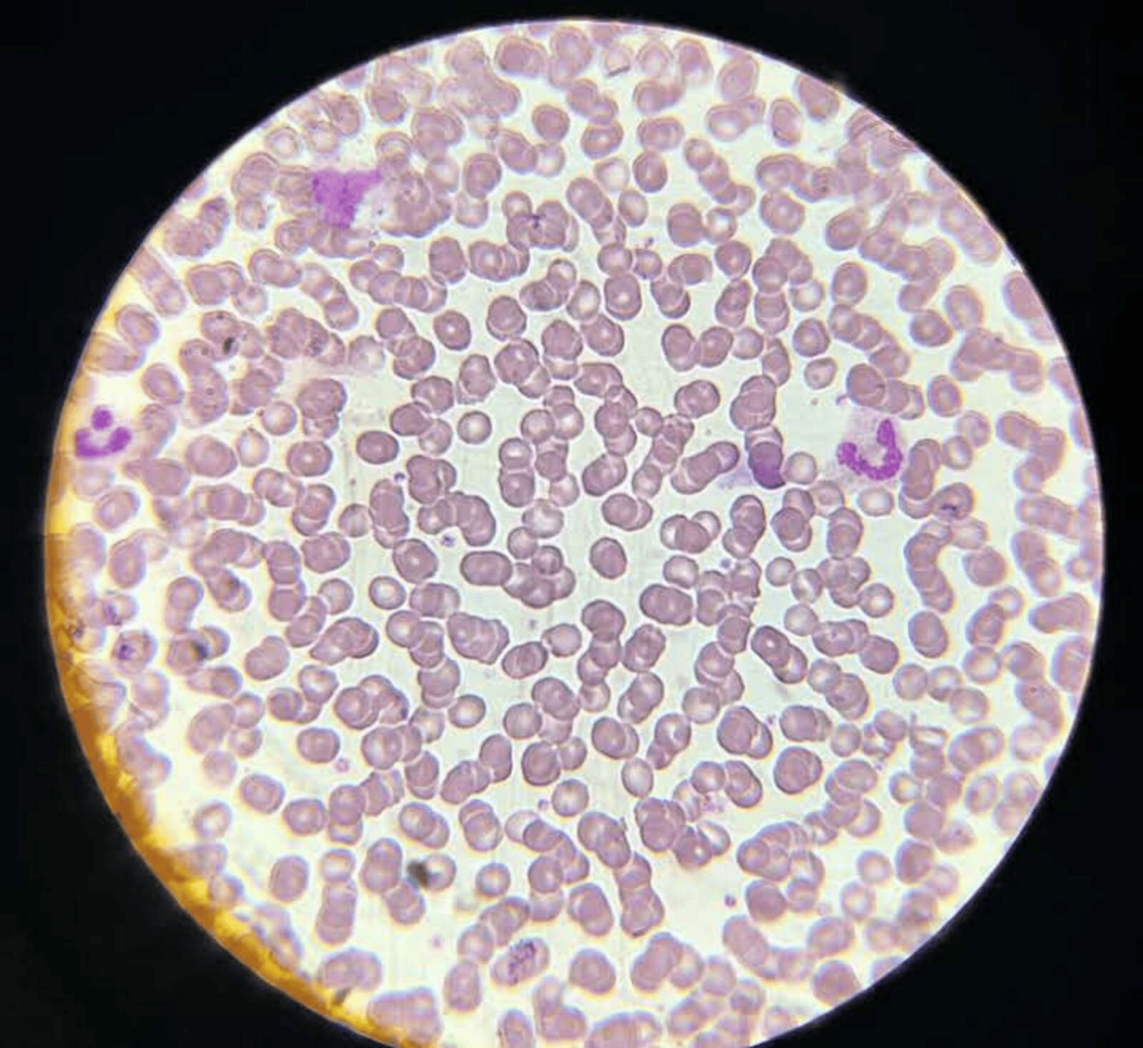
A high-power view of the peripheral smear stained with Leishman stain shows normocytic, normochromic red blood cells with increased red cell mass

The patient's blood samples were sent, and they were found to be negative for Janus kinase (JAK) mutation. The peripheral smear of the patient was suggestive of increased RBC mass with normocytic, hyperchromic RBCs (Figure 5).

To treat the condition, the patient was given injectable steroids (hydrocortisone) and pheniramine maleate and was gradually veined off. The patient was discharged after six days of treatment and was followed up after two weeks. The patient was reportedly doing well. Follow-up investigations are summarized in Table 2.

**Table 2:** Follow-up investigations of the patient after two weeks

Investigation	Observed value	Lab reference
Hemoglobin	16.1	13-15 g/dL
Total leucocyte count	4,900	4,000-11,000/cumm
Platelet	2,70,000	1,50,000-4,50,000/cumm
Mean corpuscular volume (MCV)	89.0	79-100 fL
Hematocrit	50	>45%

## Discussion

Bee sting hypersensitivity reactions are caused by a variety of immunologic pathways and can vary in severity from subcutaneous angioedema and mild urticaria to severe anaphylaxis. About 90% of cases of angioneurotic edema are caused by allergic reactions that trigger Type-1 hypersensitivity reactions [5], which, in turn, cause angioedema in a matter of minutes (histaminergic-mediated mast cell and basophil activation). On the other hand, months may pass before non-allergic angioneurotic edema is noticed due to bradykinin inhibition. One powerful vasodilatory mediator is bradykinin. In angioneurotic edema, an overabundance of bradykinin is produced, which causes mucosal and submucosal swelling [5].

Many enzymes and physiologically hazardous compounds, which include apamin, histamine, phospholipase A2, mellitin, and hyaluronidase, are present in the venom of bees [1]. Increased capillary permeability caused by hyaluronidase promotes the spread of poisons. Histamine is a substance that causes localized muscular spasms and bronchospasm by stimulating neurons, dilating blood vessels, and increasing fluid production. H4-histamine receptors impact lymphocytes, eosinophils, and mast cells, among other blood cells. Plasma bradykinin levels rise seven times more than normal during an anaphylactic attack [6].

Neurologic conditions are quite uncommon, and strokes after bee stings are among them. Additionally, bee stings have been linked to cardiovascular conditions, such as sudden myocardial infarction, in individuals with both healthy and defective coronary arteries. The production of vasoactive amines appears to have contributed to the anaphylactic shock and ventricular arrhythmia in our case. Heart involvement is the most serious and potentially fatal consequence, albeit uncommon. The initial actions after a bee sting for most patients who are referred to the emergency room are resuscitation and hydration with vascular expansion treatments. To prevent anaphylaxis, substantial dosages of corticosteroids and antihistamines are used to manage pain; in extreme situations, epinephrine is administered.

There are three types of erythrocytosis: primary, secondary, and relative. A JAK mutation causes dysregulated erythrocyte, platelet, and myelocyte formation, which is the cause of primary erythrocytosis, such as polycythemia vera. Raised erythropoietin levels and an ongoing cause of erythrocytosis are the hallmarks of secondary erythrocytosis. Relative erythrocytosis patients exhibit hemoconcentration, or a decrease in plasma volume, accompanied by a relative rise in hemoglobin [6].

The assessment of erythrocytosis ought to encompass a comprehensive medical history and physical examination. It is important to assess indicators of obstructive sleep apnea, exposure to carbon monoxide, tobacco usage, and medications (including androgenic steroids).

Hypertension and erythrocytosis without splenomegaly, leukocytosis, or thrombocytosis are the hallmarks of Gaisböck's syndrome. These patients frequently experience cardiovascular issues, which can be explained by the association between modest obesity and increased blood viscosity. Moreover, there is an increased chance of thromboembolic complications for these patients. Gaisböck linked higher stress levels to heightened hematocrit and hypertension and underlined how tense and nervous these individuals were most of the time. Patients experiencing mental stress have been shown to have hematocrit alterations along with hypertension [7,8].

Erythrocytosis has been associated with cardiovascular mortality (hazard ratio, 2.2) [9], cardiovascular morbidity (odds ratio, 1.8), and all-cause mortality (hazard ratio, 1.7). This highlights the significance of diagnosing erythrocytosis and investigating its cause. Patients with erythrocytosis had an increased risk of dying from myocardial infarction, pulmonary embolism, congestive heart failure, coronary heart disease, intermittent claudication, and stroke [10].

## Conclusions

In conclusion, this case report sheds light on the rare but significant association between bee stings and secondary erythrocytosis. Through this case, we have highlighted the importance of considering unusual etiologies in patients presenting with unexplained erythrocytosis. While bee stings are commonly associated with localized reactions, they can occasionally lead to more serious systemic complications, such as secondary erythrocytosis. Continued research into the mechanisms underlying secondary erythrocytosis following bee stings is imperative to enhance our understanding further and improve patient outcomes.
